# Coffee Drinking

**Published:** 1877-03

**Authors:** 


**COFFEE-DRINKING.**—How strong should coffee be taken? is an inquiry of much practical importance. How much should be taken at a meal? is scarcely of less moment. Coffee, like any other beverage, may wholly ruin the health; the very use of it tends to this ruin, as certainly as does the use of wine, cider, beer, or any other unnatural, stimulating drink. There is only one safe plan of using coffee, and that is, never, under any circumstances, except of an extraordinary character, exceed in quantity, frequency, or strength; take only one cup at the regular meal, and of a given, unvarying strength. In this way it may be used every day for a lifetime, not only without injury but with greater advantage than an equal amount of cold water, and for the simple reason that nothing cold should be drank at a regular meal, except by persons in vigorous health.

One pound of the bean should make sixty cups of the very best coffee. If a man takes coffee for breakfast only, one pound should last him two months, or six pounds a year.

One pound of coffee should be made to last a family of ten persons, young and old, one week. Put about two ounces of ground coffee in a quart of water, or rather divide the pound into seven portions, one for each breakfast in the week, and make a quart of coffee out of it, which will be sixty-four table-spoons. Give the youngest two table-spoonfuls and the oldest a dozen; the remainder of the one cup being filled up with boiled milk. This will give a cup of coffee sufficiently strong for all healthful purposes, for the respective ages; and for various reasons, pecuniary as well as physical, some such systematic plan as this should be adopted in every family in the land. How to make the cup of good coffee? is a third question. It is perhaps as good and as easy a plan as any to buy the coffee in the grain, pick out those that are imperfect, wash it, parch as much as will last a day or two, with your eye upon it all the time until it is of a rich brown, with no approach of black about it. Grind only enough for the day's use; grind it fine, for the greater the surface exposed to the hot water the more of the essence you will have ; pour the boiling water on the coffee, close it up, boil it ten minutes, let it stand to clear ten minutes, then use.

There are additional devices for husbanding the aroma, but as people who are so very particular about every thing they eat being done to the nicest shade, are but a shade above the brutes, and generally die twenty years before their time of inanition, of chronic diarrhea, it is not thought important to initiate the readers of this JOURNAL any further into the mysteries of coffee-making and drinking.

